# From Manual Search to Artificial Intelligence: A Methodological Framework for Developing Patient Journey Models from Electronic Health Records Using Natural Language Processing

**DOI:** 10.3390/healthcare14213768

**Published:** 2026-11-04

**Authors:** Zihan Zhang, Tara Raftery, Thirupathi Pattipaka, Petra Nathan

**Affiliations:** 1 KMK Consulting, Morris Plains, NJ 07950, USA; 2 Novartis Ireland Limited, D18T6T7 Dublin, Ireland; 3 Novartis Pharma AG, 4056 Basel, Switzerland; 4 Novartis Pharma GmbH, Evidence Generation, 90443 Nuremberg, Germany

**Keywords:** natural language processing, healthcare decision support, electronic health records, real-world data, patient journey model

## Abstract

Background: Improving care pathways for chronic conditions requires a holistic, data-driven understanding of the patient journey/ care continuum. This study introduces a novel framework to reconstruct and simulate patient journeys using medical data and probabilistic modeling. Chronic spontaneous urticaria (CSU) was selected as a use case, with first documented symptom occurrence estimated through natural language processing (NLP) and treatment cycles identified from Anatomical Therapeutic Chemical (ATC) code sequences, an approach not previously applied in this context. Objectives: To develop and evaluate a potentially scalable framework for reconstructing patient journeys and simulating care pathways in chronic diseases, with the aim of identifying and quantifying care gaps and finding opportunities to reduce delays, improve treatment initiation, and support value-based care. Methods: A Markov model was developed to simulate transitions across health states using de-identified electronic health record (EHR) data from 74,664 patients with CSU. Patient journeys were reconstructed by combining NLP-based estimation of first documented symptom occurrence with medication treatment cycles derived from ATC code sequences. The model was then used to characterize the current care pathway. Results: The analysis identified substantial delays in diagnosis and considerable variability in treatment initiation. Incorporation of NLP-derived symptom information from unstructured clinical notes increased the number of patients with a documented symptom record from 29,457 to 56,551. The interval between first documented symptom and diagnosis was 157 days (median 0) using ICD-9/10 codes only and 570 days (median 624) using ICD-9/10 codes plus NLP. Because the NLP output was not independently validated and the look-back period was limited, these values are estimates and should be read with caution. Notably, 29.7% of patients received no prescribed therapy. Conclusions: This study demonstrates the feasibility of reconstructing and simulating patient journeys using routinely collected healthcare data. Although intentionally simple, the conceptual framework may be applicable across chronic conditions, however, its implementation would require disease-specific adaptation and validation. The Markov model provides a framework for simulating targeted interventions designed to streamline care pathways, reduce delays, and optimize resource utilization in order to support data-driven decision-making, selection of targeted interventions, care pathway optimization, and the advancement of value-based healthcare.

## 1. Background

Humans often engage in implicit model-based reasoning, such as using past experiences to mentally simulate and predict traffic conditions when selecting a driving route. The situation in healthcare systems is not different. Healthcare systems can significantly improve their healthcare processes, patient experience and quality of care by understanding, analyzing, and optimizing patient pathways [1,2]. Understanding the epidemiological patient journey could improve patient care pathways, especially for chronic conditions already burdened by diagnostic delay and scattered treatment plans. The patient journey is defined as the assembly, planning, monitoring and coordination of all the steps necessary to complete the work of patient care [1,2,3,4,5], with the goal of curing patients or providing care that achieves symptom control and patient well-being. Patient journey models intend to map and visualize how the patients move through the healthcare system, from the initial experience of symptoms to diagnosis and treatment initiation, to adjustment, and follow-up. These models are increasingly used to support clinical decision-making and the design of health policies by simulating the impact of care interventions and identifying delays, redundancies, or missed opportunities [1,2].

A probabilistic patient journey model can identify barriers and bottlenecks in care delivery, supporting process optimization for patients and caregivers. To ensure clarity and applicability, the model should emphasize simplicity, robustness, and the integration of readily available, representative data. Moreover, the model should be able to capture two key dimensions of patient care: the likelihood of receiving care and the corresponding timeframe in which care is delivered. However, traditional journey models usually rely on data originating from a variety of sources, which is mostly integrated manually and can be inconsistent. They usually lack a unifying analytical framework, which makes it difficult to interpret patient pathways in a consistent and scalable manner [6]. Data extraction based on a multi-source approach using a structured literature search and combining it with clinical data and expert knowledge integration is quite fast, reliable and represents the current situation [7]. In principle, such a consensus-based approach can be used for any indication and country to incorporate country-specific data, regardless of the availability and accessibility of local real-world data sources (e.g., registries, health insurance data, electronic health records [EHR]). Nevertheless, it has several limitations and potential biases, including reliance on opinion, use of heterogeneous data sets, lack of statistical power, inconsistent data intervals, and concerns about the reliability and traceability of literature sources (e.g., market research or patient self-reported data) [8].

The increasing availability of RWD from EHRs supports the development of dynamic patient journey models [9]. Natural Language Processing (NLP) helps overcome limitations by extracting insights from both structured (e.g., ICD codes, medications and procedures codes) and unstructured data (e.g., clinical notes, symptoms) [10]. Unstructured information often holds valuable insights that structured data cannot capture. NLP has emerged as a powerful tool to mine this rich clinical text, enabling researchers to extract symptoms, disease milestones, and care patterns that would otherwise remain hidden [11,12]. Studies have demonstrated that combining NLP with RWD can reveal meaningful trends in patient trajectories, enhance phenotyping accuracy, and improve the prediction of disease progression [13,14,15]. Thus, a combination of structured data and narrative clinical details is needed to provide an accurate picture of the patient journey in the real world. However, information on treatment monitoring and symptom control is still missing to create a comprehensive patient journey model. Despite recent advancements, few studies have leveraged NLP-based methods to reconstruct patient journeys within a cohesive modelling framework [10]. Even fewer have applied this approach to chronic spontaneous urticaria (CSU), a condition marked by unpredictable symptoms, diagnostic uncertainty, and variable treatment response. CSU represents a particularly challenging case for patient journey modelling due to its complex care pathway, which often involves multiple lines of therapy, repeated clinical encounters, and delayed symptom resolution [16].

While prior CSU journey studies like that of Maurer et al. [7] have used expert input and literature-based assumptions to build probabilistic models, they often lack grounding in real-world, patient-level data. Such models, though valuable for scenario planning, may not reflect the actual diversity and variability of clinical practice. To address this gap, the current study proposes a framework for building a CSU patient journey model using a single-source EHR dataset that includes both structured and unstructured data. By applying NLP techniques to extract timelines from physician notes and aligning these with coded treatment events, we aim to develop a Markov-based probabilistic model of patient progression. This approach enables the simulation of real-life care patterns, identifying areas where interventions may be required and provides a scalable method applicable to various diseases and data systems.

## 2. Methods

### 2.1. Study Design and Data Source

We conducted a retrospective cohort study using de-identified electronic health record (EHR) data from the OPTUM^®^ database [Optum Inc. UnitedHealth Group, Eden Prairie, MN, USA], covering the period from January 2016 to December 2019. The dataset includes longitudinal clinical information from over 102 million patients across 700 hospitals and 7000 clinics in the United States. Data are HIPAA-compliant and anonymized; therefore, institutional review board approval was not required.

Patient Journey Framework (as a Markov model structure) and assumptions.

The patient journey model was implemented as a discrete-state Markov framework based on the previously published CSU patient journey structure described by Maurer et al. [7], see also supplementary information (Figure S1). The model contains five health states representing key stages of the disease pathway: Healthy, Symptomatic, Diagnosed, drug-persistent, and drug-changed.

The Healthy state represents individuals without documented CSU-related symptoms. The Symptomatic state represents patients with documented symptoms suggestive of CSU but without a formal diagnosis. The Diagnosed state represents patients with recorded CSU diagnosis codes. Drug-changed corresponds to diagnosed patients who initiated treatment but subsequently treatment change has been observed in EHR, while Drug-persistent represents patients without documented treatment change following treatment initiation. (In this manuscript the terms Treated-stable and Treated-adjusted represent proxy treatment states based on observed treatment transitions rather than directly observed clinical disease control.)

These states were selected because they reflect clinically relevant milestones along the patient pathway and correspond to stages that can be identified within routinely collected EHR data.

Transition probabilities were estimated directly from observed patient-level data within the OPTUM^®^ EHR database. For each transition, the proportion of patients moving from one state to the next was calculated using the corresponding cohort counts obtained from structured diagnoses, NLP-derived symptom information, and treatment records. Durations between transitions were estimated from observed time intervals and summarized using descriptive statistics including mean, median, and standard deviation. Transition probabilities were estimated from pooled observations across 2016–2019 and therefore represent average transition behavior over the observation period under a time-homogeneous (stationary) assumption. This assumption was not formally tested. Treatment recommendations for CSU evolved during the observation period, in particular through the increasing uptake of omalizumab, and the distribution of index dates declines across the period. Any temporal drift would be averaged out in the pooled estimates rather than detected; a period-stratified sensitivity analysis would be required to test the assumption directly.

Transitions are characterized by both likelihood (e.g., diagnosis rate, treatment initiation rate) and timing (e.g., time to diagnosis, time to treatment change). This dual-dimensional structure enables simulation of care pathways and identification of bottlenecks.

The model uses a one-day cycle length because all events were recorded using date-level information within the EHR database. This approach allows direct incorporation of observed event timing without additional temporal aggregation. The initial state distribution was derived from the observed patient population. Individuals entered the model according to the earliest disease-related event identified within the available EHR record. Because the focus of the analysis was reconstruction of the patient journey among patients with CSU, the symptomatic and diagnosed states served as the primary entry points into the model.

Right censoring was present because patient follow-up was limited to the observation period from January 2016 through December 2019. Patients without a subsequent documented event before the end of follow-up contributed information up to their last available record. No extrapolation beyond observed follow-up was performed.

Handling of missing data is described in Section 2.2.7. No statistical imputation was performed. Analyses were based on available documented events and complete cases for each transition parameter.

No external validation dataset was available. Model validation therefore focused on internal consistency checks and assessment of the clinical plausibility of estimated transition probabilities and timelines against published CSU patient journey evidence.

The objective of the Markov component was to approximate aggregate patient flows rather than predict individual disease evolution.

### 2.2. Data Extraction Workflow

The translation of data into the existing patient journey model requires a careful and systematic approach (Table 1). Our exploratory methodology comprises several interconnected steps designed to extract maximum value from structured and unstructured electronic health record data as well as metadata. Our approach integrates structured claims data with unstructured clinical notes. For structured data elements (ICD codes, prescription records, demographic information), we employed direct database queries with standardized terminologies to ensure consistent data representation. For unstructured data within clinical notes, we leveraged the natural language processing (NLP) capabilities provided by Optum^®^, which extracts critical symptom information that would otherwise remain hidden within physician narratives. Metadata (time between care steps, time of diagnosis. frequency of measurements) were extracted to better interpret treatment pattern and patient outcomes without needing full patient details.

This methodology enhances model relevance, improves interpretability and allows to uncover treatment pattern and estimate patient outcomes in order to support more informed decision-making in clinical and operational contexts (Figure 1).

The integration process follows a sequential workflow:

1. Initial extraction of structured data elements using standardized query language
2. Secondary extraction of unstructured data through NLP processing of clinical notes
3. Temporal alignment of extracted data points to create a chronological patient pathway
4. Data harmonization to resolve inconsistencies between structured and unstructured sources
5. Validation of extracted data through cross-referencing between data modalities

An overview of the data to be extracted is provided in Table 1.

**Table 1:** Data input needed to build the Markov model for the CSU patient journey (see Figure S1 and [7]).

Input data	Explanation
Prevalence	Prevalence rate of CSU
Diagnosis rate	Patients with CSU diagnosis
Diagnosis time	Time between first documented symptom occurrence related to CSU and first CSU diagnosis (=index date, see Figure 2)
Treatment start rate	Number of patients with recorded medical therapy for CSU
Treatment start time	Time between first CSU diagnosis and first documented CSU-related prescriptions
Treatment change rate	Number of patients with multiple documented CSU-related prescriptions
Time to treatment change	Time (median) between different drug prescriptions
Never diagnosed patients	Patients with CSU symptoms without physician-diagnosis of CSU
Never treated patients	Diagnosed Patients without observed disease-specific treatment
Healthy	Patients without CSU symptoms
Symptomatic	Patients with CSU symptoms without being diagnosed
Diagnosed	Patients with physician-diagnosed CSU
Drug-persistent	Diagnosed CSU patients with at least one documneted CSU-related prescription and no subsequent change in drug, as prescribing-pattern state
Drug-changed	Diagnosed CSU patients with at least one change of the ATC/NDC code relative to the preceding CSU-related prescription, as prescribing-pattern state

#### 2.2.1. Definition of No Recorded Therapy

Patients were classified as having no recorded therapy if no CSU-related prescription record associated with the predefined ATC or NDC codes was identified during the observation period following the index diagnosis. This definition reflects the absence of documented physician-prescribed CSU treatment within the available OPTUM^®^ EHR data and should not be interpreted as confirmation that no treatment was received.

#### 2.2.2. Natural Language Processing for Symptom Identification

To reconstruct the symptomatic phase of the patient journey, we supplemented structured EHR data with symptom information extracted from unstructured clinical documentation using the NLP functionality provided within the OPTUM^®^ EHR database. Although the proprietary details of OPTUM^®^’s NLP implementation are not publicly disclosed, the system fundamentally operates by identifying medical concepts within free text and mapping them to standardized terminology. Negated findings and unrelated clinical concepts were excluded whenever identifiable within the available NLP output structure.

The objective of using NLP was not to develop or validate a novel symptom-extraction algorithm, but rather to leverage routinely available NLP-derived variables from a large EHR database to augment patient journey reconstruction. Specifically for symptom extraction, the NLP system analyses clinical notes for mentions of CSU-related symptoms, such as wheals, itching and angioedema. The system accounts for the linguistic complexity of clinical documentation by recognizing various forms of symptom descriptions, including (Table S1):

1. Direct symptom mentioned (“patient reports wheals”)
2. Temporal qualifiers (“recurring hives for past 3 months”)
3. Severity indicators (“severe pruritis”)
4. Anatomical locations (“angioedema affecting lips”)
5. Negated findings (“no evidence of wheals today”)

The extracted symptom information is then structured into a standardized format containing the symptom term, attributes (severity, duration), sentiment (positive/negative finding), anatomical location, and mapped medical concept (Figure S2). This structured representation allows us to integrate previously inaccessible clinical information into our patient journey model.

Only symptom records classified as positive findings within the NLP output were retained for analysis. Records classified as negated findings were excluded. Historical symptom status, experiencer status, and assertion categories beyond the sentiment variable were not available in the NLP output provided to investigators; therefore, symptom onset was operationally defined as the earliest positive symptom mention available within the EHR record.

Importantly, access to the underlying NLP algorithms, training datasets, and performance metrics was not available to the investigators because the NLP functionality is proprietary to OPTUM^®^. Consequently, independent validation of the NLP extraction process through manual chart review or calculation of performance metrics such as sensitivity, specificity, precision, and recall was not feasible within the scope of this study.

The NLP search was applied within the cohort of ICD-10-diagnosed CSU patients and restricted to the pre-diagnosis period. It covered CSU-related terms and unspecific dermatological terms including rash, itching, pruritus and angioedema, with anatomical localization used to restrict the results towards CSU-typical sites. The complete list of terms are provided in Supplementary Table S2. Negated findings were excluded; assertion, historical and experiencer status were not available in the NLP output provided to the investigators, so the extracted mentions cannot be regarded as confirmed CSU-specific documentation.

#### 2.2.3. Definition of Disease Specific Codes

Disease-specific diagnosis codes for CSU were used based on the International Classification of Diseases (ICD) coding system. Both the 9 th edition (ICD-9: 708.1, 708.8, 708.9, 995.1) and the 10th edition (ICD-10: L50.1, L50.8, L50.9, T78.3) were used to account for the long observation period.

In addition, in accordance with guideline recommendations [17], specific drug codes for CSU treatment, identified by National Drug Codes (NDC) and Anatomical Therapeutic Chemical (ATC) codes, were used to analyze prescribed CSU-specific treatments.

#### 2.2.4. Data Extraction

Patients diagnosed with CSU during the observation period from January 2016 to December 2019 were identified using ICD-9 and ICD-10 codes. The index date was defined as the first report of a CSU diagnosis within this period. Diagnosed patients were defined as those with at least two ICD codes, separated by at least 6 weeks but no more than 52 weeks, in either order.

From this cohort, a retrospective assessment of first documented symptom occurrence was performed to identify ‘symptomatic patients’. This was done as follows:

(A) Symptomatic patients were identified from structured data: The OPTUM EHR diagnosis table was searched for the first occurrence of ICD codes (R21, L29.9, T78.3);

(B) Symptomatic patients (sentiment positive) using unstructured data from the OPTUM EHR NLP_SDS table: Variables used included term, attribute, sentiment, location and concept (see Table S1, Supplement). The time to CSU diagnosis (diagnosis time) was examined based on the date of first documented symptom occurrence in the patients. NLP was applied as provided via OPTUM^®^ EHR.

Appropriate ATC and NDC codes were used to assess medical therapy within the diagnosed patient pool. Treatment initiation was defined as the first documented CSU-related prescription occurring after the index diagnosis. To characterize treatment as change over time, ATC and NDC codes were mapped and ordered chronologically for each patient. Treatment sequences were then derived from the order of documented prescriptions during the observation period.

A treatment change was defined as the occurrence of an ATC/NDC code different from the immediately preceding CSU-related medication record. Because detailed information on treatment intent, dosage adjustment, prescription duration, refill status, and medication discontinuation was not consistently available, treatment transitions could not be further classified into the different treatment management strategies (as escalation, switching, cycling etc.). This approach was intended to identify treatment change and characterize prescribing patterns at the population level. Because cycling between therapies within the same drug class is common in CSU management, drug change was assessed at the individual drug level rather than solely at the drug-class level. Consequently, treatment pathways should be interpreted as sequences of documented prescribing events rather than confirmed medication-use episodes.

Treatment gaps were not assessed because prescription duration and treatment discontinuation data were not sufficiently complete to support reliable estimation.

Drug-change was used as a proxy a change in prescription within the patient journey model.

For a schematic representation of the applied definition for data extraction and cohort identification see Figure 2.

To increase the likelihood that our cohort comprised newly diagnosed cases rather than patients receiving ongoing treatment for pre-existing disease, a washout period was applied. This period was defined as the one-year interval preceding the index date (first CSU diagnosis), during which patients had no record of CSU-specific treatments, as identified by our selected ATC codes. These codes corresponded to the drug groups most commonly prescribed for CSU management, including H1-antihistamines, higher-dose H1-antihistamines, biologics (e.g., omalizumab), and other adjunctive therapies such as leukotriene receptor antagonists.

Since the OPTUM^®^ EHR lacks any information on CSU symptom control through clinical text notes or patient-reported outcomes (PROs) like the Urticaria Control Test (UCT), Dermatology Life Quality Index (DLQI), or Urticaria Activity Score over 7 days (UAS7), we applied a proxy to estimate the number of patients who reach stable disease status with physician-prescribed treatments. Our methodology uses treatment transitions as observable proxies for treatment change. Specifically, a patient with two different ATC codes would be considered drug-changed on the first prescribed drug (1 ATC code) but drug-persistent on the second drug (2 ATC codes). However, this approach does not account for label-recommended treatment changes, such as dose escalation.

#### 2.2.5. Data Analysis

The approach is primarily descriptive and model-based, with a focus on characterizing time intervals and transition probabilities along the patient journey, rather than testing specific hypotheses regarding differences between groups. The data reflects direct observations from the entire population meeting our inclusion criteria, rather than a sample requiring inferential statistical methods.

In this context, the clinical relevance of observed time intervals (e.g., 570 days from first documented symptom occurrence to diagnosis) can be interpreted directly, without reliance on *p*-values. This descriptive, model-based approach focuses on characterizing time intervals and transition probabilities across the patient journey, without testing group differences. As the data represents the full eligible population, inferential statistics are not required. Standard time-to-event analyses were performed, allowing direct interpretation of clinically relevant patterns.

#### 2.2.6. Data Integration

Data integration involves merging the cleansed and transformed data from both OPTUM^®^ EHR claims and NLP tables into the existing analytical model (Figure 2). This step ensures a single, comprehensive view of the patient journey by combining structured claims data with unstructured symptom information, resulting in a holistic dataset for subsequent analysis. For the Markov model component, we employ a deterministic mathematical framework based on observed transition probabilities, rather than statistical inference. The model is designed to simulate population dynamics across health states using real-world data, rather than to test statistical hypotheses.

#### 2.2.7. Handling of Missing Data

A significant challenge in real-world EHR-based research is the presence of missing data. In our study, we observed missing data patterns across several key variables, including first documented symptom occurrence information (24.26% of diagnosed patients) and treatment data (29.7% of patients had no recorded ATC code). We adopted a complete case analysis approach for the following reasons:

1. Methodological appropriateness: No statistical imputation was performed, and the analyses are based on available documented events. Missing records in the EHR may represent either an absence of care or an absence of documentation. For treatment records, for example, a patient may have received no physician-prescribed medication, may have relied on self-medication, or may have filled the prescription outside the captured data source. The direction of this ambiguity cannot be determined from the available data: if missing records reflect an absence of care, our estimates describe care patterns accurately, whereas if they reflect incomplete documentation, treatment rates and resource use are underestimated.
2. Preservation of data integrity: By excluding cases with missing data from specific analyses rather than imputing values, we maintain the integrity of the observed data. This approach ensures that our findings reflect actual, documented clinical pathways rather than relying on statistical approximations.
3. Transparency in reporting: We explicitly report the number of cases excluded due to missing data for each analysis, enabling readers to evaluate its potential impact on our findings. This transparency is crucial for interpreting the generalizability of our results.
4. Indication-specific considerations: For CSU, patterns of missing data may be associated with disease severity or with healthcare utilization patterns. Imputation could obscure such differences; it could equally mask systematic under-documentation. We therefore report the proportion of excluded records for every parameter rather than imputing them.
5. It is important to note that our complete case analysis approach introduces the potential for selection bias if the mechanism underlying the missing data is not random. For treatment data, absence of documentation may reflect either true absence of therapy or incomplete treatment capture.

#### 2.2.8. Data Validation and Clinical Plausibility Checks

To ensure the accuracy and clinical relevance of the extracted data, we implemented a multi-step validation process. This included both technical cross-validation between structured and unstructured data sources and clinical plausibility checks based on demographics and disease knowledge.

First, symptom timelines, diagnosis events, and treatment records extracted from structured and unstructured data sources were assessed for internal consistency and temporal plausibility. Implausible sequences and inconsistent records were flagged and reviewed during data processing.

Second, because independent validation of the proprietary NLP algorithm was not feasible, validation focused on the clinical plausibility of the resulting patient journey metrics rather than the NLP component itself. Extracted variables were compared with published real-world evidence, guideline-based expectations, and previously published CSU patient journey models [7]. The objective was to assess whether the integrated EHR-derived framework reproduced known characteristics of CSU care pathways, such as prolonged diagnostic delays, patterns of treatment initiation, and treatment change observed in routine clinical practice.

## 3. Results

### 3.1. Demographics and Background of the Patient Population

90,091 unique patients with diagnosed CSU were identified at the index date, of which 74,664 (82.9%), were found to be unique patients after applying the washout period. Overall demographics were as follows: 62% female, 38% male, with a mean age of 32.72 years. The ethnic composition was 54% of patients having no reported ethnicity, 22% identified as white, 10% as hispanic and 7% as black.

### 3.2. Time from First Documented Symptom Occurrence to Diagnosis

Of the 74,664 unique patients in the diagnosed cohort, data from 56,551 patients (75.74%) contained documented symptom information enabling calculation of symptom-to-diagnosis intervals. This absence of pre-diagnosis symptom documentation could represent patients who received initial care outside the database’s capture area, those with sudden documented symptom occurrence prompting immediate diagnosis, or cases where symptoms were simply not documented in accessible clinical notes.

Following the cohort of 56,551 unique patients (75.74% of the initial cohort) we found that mean time from first documented symptom (coded and free-text symptoms) occurrence to diagnosis was 570 days (SD 395 day), with a median of 624 days (Table 2), indicating considerable variation in the time patients waited before receiving a diagnosis. Among these patients, 6800 (12.0%) had a same-day interval, meaning that the first documented symptom and the diagnosis were recorded on the same date. These intervals reflect documentation practice rather than an absence of diagnostic delay; records without any documented symptom date were excluded and are reported separately (Table 2). Among patients whose symptoms were identified exclusively through structured ICD-9/10 coding, the mean interval between first documented symptom and diagnosis was only 157 days. In contrast, when NLP-derived mentions from unstructured clinical notes were added, the mean observed interval to 570 days. The difference between the two figures should not be read as a longer or a more accurate interval: the NLP search included unspecific dermatological terms and could not exclude historical or unrelated mentions, so every additional finding precedes the diagnosis and lengthens the estimate. The added value of NLP in this analysis lies in the number of patients with an identifiable symptom record, which arose from 29,457 to 56,551, and in the reduction of patients without any symptom documentation from 45,207 to 18,113 (Table 2).

For descriptive comparison, the EHR-derived state proportions and transition timings were set alongside the estimates of the published CSU patient journey model by Maurer et al. [7] (Supplementary Tables S3 and S4). The overall treatment rate was similar (70.3% vs. 65.3%), as were the proportions of drug-persistent patients on the second (63% vs. 63.2%) and third drug (67.7% vs. 67%), whereas the proportion with a first drug change was higher in the EHR data (47.5% vs. 25%); time from symptom onset to diagnosis was shorter (570 days vs. 2 years), and time to treatment initiation (101 days vs. 3 months) was comparable, while intervals between therapy steps were shorter in the EHR data (Table S4). The proportion of patients with a documented first treatment change was higher in the EHR data than in the model of Maurer et al. (47.5% vs. 25%, a difference of 22.5 percentage points). This difference is likely to reflect the operational definition: in our analysis, treatment change was assessed at the level of the individual drug, so a change within the same therapeutic class was counted as a treatment change, whereas the comparison model was based on expert-derived treatment states. A difference in clinical practice between the US and German settings may also contribute, but the available data do not allow the two effects to be separated. Because our states are prescribing-pattern states and not clinical disease states, this comparison is descriptive and not a validation.

### 3.3. Time from Diagnosis to Treatment

In the patient cohort, 29.7% had no documented CSU-related prescription record within the available EHR dataset. Patients classified as having no recorded therapy had a mean age of 34.7 years and were predominantly female (63% female, 37% male). Race and ethnicity information were incomplete, with 54% of patients having no specified race; among those with available information, 21% were classified as White, 15% Hispanic, and 9% Black. These patients were classified as having no recorded therapy for the purpose of patient journey reconstruction.

Within the patient group who have documented drug-prescription most patients received one (36.9%), two (21.1%) or three (8.4%) physician-prescribed drugs to treat CSU.

Among patients who started treatment, the mean time from diagnosis to first treatment was 101 days (SD 208) and the median was 0 days. The standard deviation of 208 days suggests substantial variability in treatment-initiation timing across patients (Table 2). 27.1k patients had a same-day interval, representing patients who had diagnosis and treatment coding at the same day.

Among patients with a documented second treatment, the mean interval from diagnosis was 194 days (SD 244; median 99). For the third treatment, the mean was 282 days (SD 308; median 157), and for the fourth, 331 days (SD 326; median 209). The number of patients with an evaluable interval decreased with each treatment line (Table 2). Same-day intervals and records without a documented interval are reported separately for each line.

### 3.4. Observed Disease Status

All patients had at least one additional ICD-10 coding for CSU following treatment exposure. While this finding indicates subsequent CSU-related healthcare utilization, diagnostic coding alone does not permit conclusions regarding disease activity, symptom burden, clinical control, or remission status.

### 3.5. Summary of Extracted Data to Build the Final CSU Patient Journey Model

A summary of extracted EHR data is shown in Figure 3. The data from this decision tree were then transferred into the proposed Markov model to define transition probabilities and transition times between the health states. Finally, based on the simulation, Figure 4 displays the current patient journey for patients with chronic spontaneous urticaria in the US population, based on OPTUM^®^ EHR data.

While intervention scenarios were not formally evaluated in the present study, the framework could be extended in future work to simulate and compare alternative care pathway interventions.

## 4. Discussion

A growing body of work highlights the potential of extracting longitudinal data from electronic health records (EHRs) to construct quantitative patient journeys, enabling a more objective and data-driven understanding of healthcare processes [18,19]. Thes approach is increasingly used to inform resource planning, process optimization, and the design of targeted interventions as it allows the assessment of healthcare system architecture and patient flow in a real-world context rather than relying on idealized models [19].

However, despite this momentum, the field remains constrained by the lack of standardized metrics, harmonized definitions, and robust comparative methodologies, which limit cross-study comparability and scalability [18,19].

In this context, our study provides a novel contribution by introducing an integrated methodological framework that combines structured data, unstructured clinical narratives, and metadata within a unified probabilistic modeling approach. This enables a more granular and temporally resolved reconstruction of real-world care pathways compared to prior models that rely on expert opinion, aggregated evidence, or single data modalities. Recent studies have demonstrated the value of integrating multiple EHR data modalities to characterize patient trajectories and disease progression [13,20].

A key innovation of this work lies in the systematic use of metadata—such as timing between clinical events and frequency of healthcare interactions—to reconstruct longitudinal dynamics and care sequencing without requiring complete clinical records [20]. This allows for a more comprehensive representation of patient trajectories and enhances the interpretability of real-world care patterns.

A key methodological finding of this study was the contribution of NLP-derived symptom information to patient journey reconstruction. The addition of symptom mentions extracted from unstructured clinical notes increased the number of patients with a documented symptom record from 29,457 to 56,551, substantially expanding the population from whom symptom trajectories could be assessed (Table 2). This should be read as a gain in completeness rather than in accuracy, because the NLP-derived mentions were not confirmed as CSU-specific and the extraction could not be validated.

The findings derived from this approach highlight substantial inefficiencies in CSU care delivery. The observed delay in CSU of nearly two years between first documented symptom occurrence and diagnosis suggests persistent gaps in disease recognition, diagnostic pathways, or care continuity in real world which is well known from literature [21]. Similarly, the high proportion of no recorded therapy indicates a critical disconnect between diagnosis and therapeutic intervention, potentially reflecting barriers related to access, physician decision-making, or patient behavior. However, some findings should be interpreted cautiously like the 29.7% of patients without documented CSU-related prescription records following diagnosis. Absence of a documented prescription within the EHR does not necessarily indicate absence of treatment. Patients may have used over-the-counter antihistamines, received treatment outside healthcare organizations contributing data to the OPTUM^®^ database, obtained medications through alternative care settings, or experienced incomplete documentation of prescribing activity within the EHR system. Consequently, the proportion classified as having no recorded therapy may overestimate the true proportion of untreated patients.

Demographic characterization of this subgroup showed a distribution broadly like that of the overall CSU cohort, suggesting that the absence of documented therapy was not confined to a single demographic subgroup. Nevertheless, the available data did not permit detailed analyses by insurance status, healthcare setting, or geographic region. Future studies should investigate these factors to better distinguish potential care gaps from documentation-related artifacts.

Differences between the EHR-derived and published estimates may reflect (i) different health systems (Germany vs. US) and reimbursement rules, (ii) different data sources (expert-based and literature-derived inputs vs. documented prescribing events), (iii) different operational definitions of Drug-change/ treatment adjustment, and (iv) the inclusion in the EHR data of prescribing events that do not represent clinical treatment failure. The comparison is therefore descriptive and was not intended as external validation.

Chronic spontaneous urticaria presents a broader challenge commonly encountered in several chronic diseases, including migraine, chronic pain disorders, and selected dermatological and rheumatological conditions, where disease burden is not routinely captured through a single objective biomarker. Instead, assessment of disease activity and treatment response relies largely on validated patient-reported outcome measures. Because such measures were unavailable in the present EHR dataset, direct evaluation of disease control was not feasible. Consequently, treatment transitions were used as observable surrogate indicators of treatment adjustment, while acknowledging that these measures do not represent validated assessments of clinical disease activity.

Our framework builds on and extends prior work in patient journey modeling across multiple disease areas. Previous studies in chronic spontaneous urticaria, respiratory diseases, and diabetes have demonstrated the value of structured patient pathway models for identifying care gaps and optimizing healthcare delivery [7,22,23]. Conceptually, our approach aligns with these efforts through a stepwise patient flow framework, in which populations are progressively refined across key stages of the care pathway. Thus, based on documented use cases the structure of the patient journey model and the health states applied have been shown to be applicable and generalizable across chronic conditions, supporting its use as a standardized approach for longitudinal patient journey analysis.

Our work further advances the field by using NLP to extract clinically meaningful information from unstructured data and integrate it with structured EHR elements, consistent with recent AI-driven patient trajectory studies that support this methodological approach [24].

This combined approach enhances cohort identification and enables more accurate reconstruction of disease timelines, particularly in conditions where symptom documentation precedes formal diagnosis. Together, the integration of multimodal data and probabilistic modeling represents a shift from fragmented data analysis toward a cohesive and scalable framework for patient journey reconstruction and care gap analysis.

The framework could be used to evaluate alternative care scenarios which may support luation of alternative care scenarios, including earlier diagnosis, optimized treatment, and improved resource allocation. These insights provide actionable opportunities for healthcare systems to identify bottlenecks, reduce delays, and design more efficient and patient-centered care pathways.

A particular strength of the framework is its ability to integrate not only structured diagnoses and treatment records and unstructured clinical narratives, but also metadata describing the timing and frequency of healthcare interactions. This approach might be transferable to other countries and indications as the underlying methodological principles are not disease-specific and may therefore be applicable across a broad range of chronic conditions.

Overall, this study demonstrates that integrating artificial intelligence with longitudinal EHR data—across structured, unstructured, and metadata domains—enables a data-driven reconstruction of patient journeys. By moving beyond descriptive approaches toward dynamic and quantitative modeling, this framework may offer a robust foundation for understanding and optimizing real-world healthcare delivery.

While the framework was developed using chronic spontaneous urticaria as a use case, extension to other disease areas should not be assumed without additional adaptation. Symptom identification strategies, documentation patterns within EHR systems, and treatment sequences vary considerably between conditions and may require disease-specific customization and validation before implementation.

The Markov framework assumes that future transitions depend only on the current state and not on the complete sequence of previous events. This assumption represents a simplification of real-world CSU management, where treatment history, disease duration, disease severity, physician preferences, and previous treatment response may influence subsequent treatment decisions. The present model was intentionally designed as a parsimonious framework to support patient journey reconstruction using routinely available EHR data. While this simplification improves interpretability and scalability, future work may explore semi-Markov or history-dependent modeling approaches that allow incorporation of treatment history and longitudinal disease trajectories.

Future work may incorporate probabilistic sensitivity analyses and uncertainty estimates around transition parameters.

Future implementations should incorporate validated disease severity and patient-reported outcome measures whenever available.

## 5. Limitations

Several limitations remain. EHR data lack standardization across sites, and key variables relevant to CSU care, such as dosage titration, step-up therapy, treatment adherence, and patient-reported outcomes, were either absent or inconsistently recorded. As a result, our use of treatment sequences as a proxy for disease control may lead to misclassification. While such assumptions are clearly acknowledged, they underscore the need for validation through clinical input and, ideally, linkage with additional real-world datasets [25,26]. Moreover, the absence of standardized patient-reported outcomes limited our ability to directly assess symptom burden and treatment response. EHR also fails to capture longitudinal care across settings, resulting in fragmented views of the patient journey. Furthermore, medication records in the OPTUM^®^ EHR primarily reflect prescribing activity documented by healthcare providers and should not be interpreted as confirmation of medication dispensing, adherence, or actual patient use. Consequently, the reconstructed treatment pathways represent documented treatment decisions rather than verified treatment exposure. Some treatment modifications, discontinuations, or interruptions may therefore not be fully captured within the available data.

Because treatment history may influence future treatment changes, the Markov assumption may not fully capture the complexity of CSU management pathways. Consequently, estimated transition probabilities should be interpreted as population-level approximations rather than patient-level predictions.

A limitation of the study is the reliance on NLP-derived variables generated through a proprietary platform whose internal algorithms are not publicly available. Consequently, independent replication of the symptom extraction process is limited.

Accordingly, estimates of symptom-to-diagnosis intervals may be influenced by the performance characteristics of the underlying NLP extraction process.

The NLP-derived symptom mentions were not confirmed as CSU-specific. The terms applied are listed in Supplementary Table S2 and include unspecific dermatological terms as well as pain and sleep problems. Although the search was restricted to the diagnosed cohort and to the pre-diagnosis period, mentions relating to other conditions or to earlier, unrelated episodes within the same record could not be excluded, and assertion and historical status were unavailable. Any such mention moves the first symptom date earlier and therefore lengthens the interval. Combined with the fact that the NLP component is proprietary and could not be validated against human-annotated data, we report the interval for two symptom definitions and describe the NLP-based value of 570 days as an upper estimate rather than as the duration of diagnostic delay.

The definition of treatment change represents a simplified operational approach based on observed ATC/NDC transitions. As detailed information regarding dosage modification, refill status, treatment discontinuation, and physician intent was unavailable, the framework could not distinguish between treatment escalation, switching, cycling, augmentation, or coding-related prescription changes. Consequently, estimated treatment-transition frequencies and timings should be interpreted as descriptive measures of prescribing patterns rather than precise representations of therapeutic decision-making. Future analyses using datasets with more granular treatment information may enable formal sensitivity analyses of alternative transition definitions.

To address these limitations, it is advisable to incorporate direct observation and collaboration with patients and clinical staff when investigating patient journeys. This approach can reveal insights that are often missing from routine healthcare data. In fragmented healthcare systems, obtaining integrated information on patient visits across providers and locations remains challenging. Future research could focus on developing advanced tokenization algorithms to link patients across multiple datasets, thereby improving our understanding of patient journeys.

We acknowledge that excluding patients with missing information may have introduced selection bias.

Transition probabilities were estimated from pooled 2016–2019 observations under a time-homogeneous (stationary) assumption. We did not formally test temporal drift, and the assumption should therefore be regarded as a modelling choice rather than a verified property. Treatment recommendations for CSU evolved during the observation period, particularly through the increasing uptake of omalizumab, and the distribution of index dates declines across the period (2016: 24,430; 2017: 21,608; 2018: 18,346; 2019: 10,280). Because the observation window ends on 19 November 2019, it also precedes the COVID-19 period, so telehealth-related changes in care delivery are not represented. Any temporal drift would be averaged out in the pooled estimates rather than detected. A period-stratified sensitivity analysis comparing 2016–2017 with 2018–2019, which the present analysis did not include, would test the assumption directly and is recommended for future work with an extended follow-up period.

A substantial proportion of records was excluded from individual analyses: 29.7% of diagnosed patients had no recorded CSU-related prescription and 24.26% had no documented symptom date. This complete-case approach may introduce selection bias, and the direction is not determinable from our data. If the missing records reflect an absence of care, the estimates describe care patterns accurately. If they reflect incomplete documentation, treatment initiation rates and time-to-treatment estimates are biased and the extent cannot be quantified. Because the two mechanisms cannot be distinguished in an EHR database without linkage to external prescribing data, the results should be read as describing documented care rather than all care delivered.

Baseline characteristics were not systematically compared between patients with and without an evaluable record, and such a comparison should be part of future work. The characteristics available for patients without a recorded therapy were a mean age of 34.7 years, 63% female, and 54% without specified race.

Same-day intervals (first and second event documented on the same date) are reported separately for each parameter, because they reflect documentation structure rather than an absence of delay and may therefore underestimate the true interval.

A like-for-like comparison with the published patient journey model [7] was not possible, because the definitions differ, for treatment change, which we assessed at the drug level while the comparison model used expert-derived treatment steps. A comparison using a class-level or step-based definition was not performed and would be required to separate a definitional artefact from genuine differences in practice between the US and German settings.

## 6. Conclusions

The methodology described has the potential to automate data extraction from real-world datasets. This in turn facilitates the rapid and cost-effective development of patient journey models across indications and countries. In addition, the use of artificial intelligence can reveal hidden patterns and information.

These findings highlight the need for greater consistency in care, improved tracking of treatment timelines, and earlier escalation of therapy when appropriate. Such measures could improve patient outcomes in the management of chronic diseases.

In particular, NLP adds critical value by enabling the transformation of unstructured clinical notes into structured data, which improves data completeness and accuracy. By allowing the application of inclusion and exclusion criteria to physician narratives, NLP ensures more precise cohort selection and supports higher-quality evidence generation. This enhances the reliability and interpretability of patient journey models built from electronic health records. Importantly, these advantages are transferable to other disease areas, reinforcing the broader applicability of this methodological framework.

## Figures and Tables

**Figure 1:**
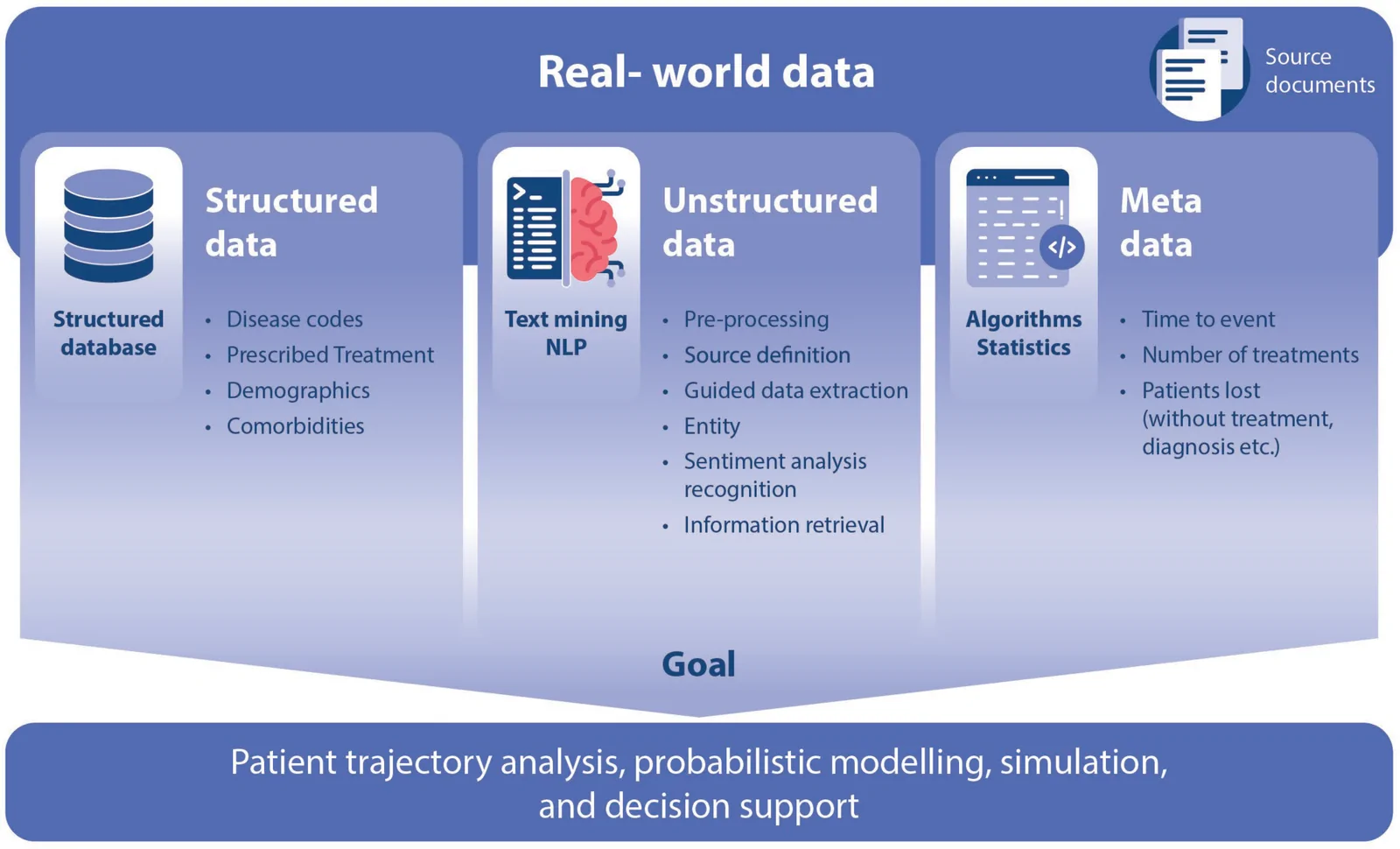
**Conceptual framework for generating quantitative patient journeys from real-world data.** The framework combines structured healthcare data, unstructured clinical information extracted through NLP, and derived journey metadata to characterize patient pathways across the continuum of care. Extracted journey parameters are subsequently used for patient trajectory analysis, probabilistic modeling, simulation of care pathways, and healthcare decision support.

**Figure 2:**
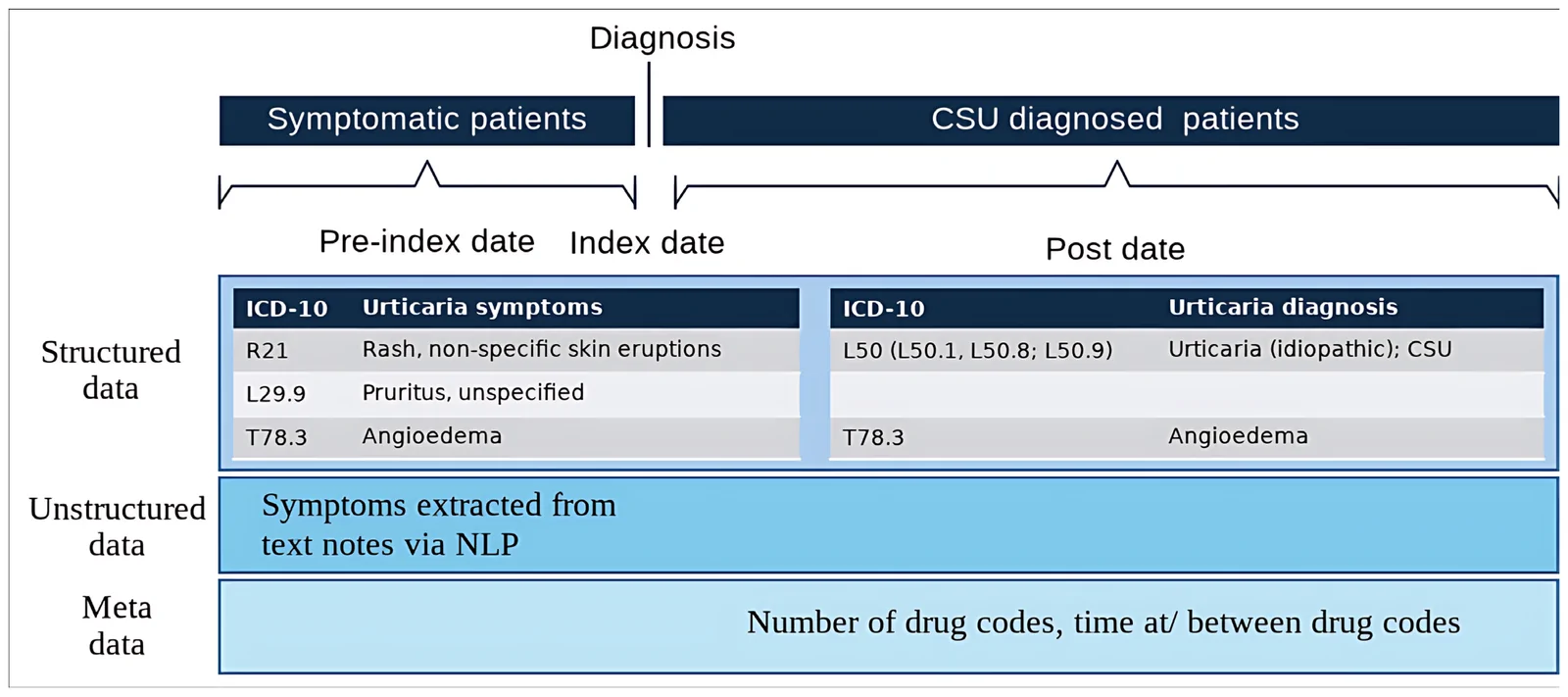
**Data elements used for the reconstruction of the CSU patient journey.** Structured diagnostic codes, NLP-derived symptoms from clinical notes, and longitudinal medication records were integrated to identify symptomatic and CSU-diagnosed patients and to characterize care trajectories. Symptom-based identification relied on ICD-10 codes for rash, pruritus, and angioedema, whereas confirmed CSU cases were identified using urticaria diagnosis codes. Medication utilization was assessed through the number and temporal sequence of drug codes, providing input parameters for the quantitative patient journey framework.

**Figure 3:**
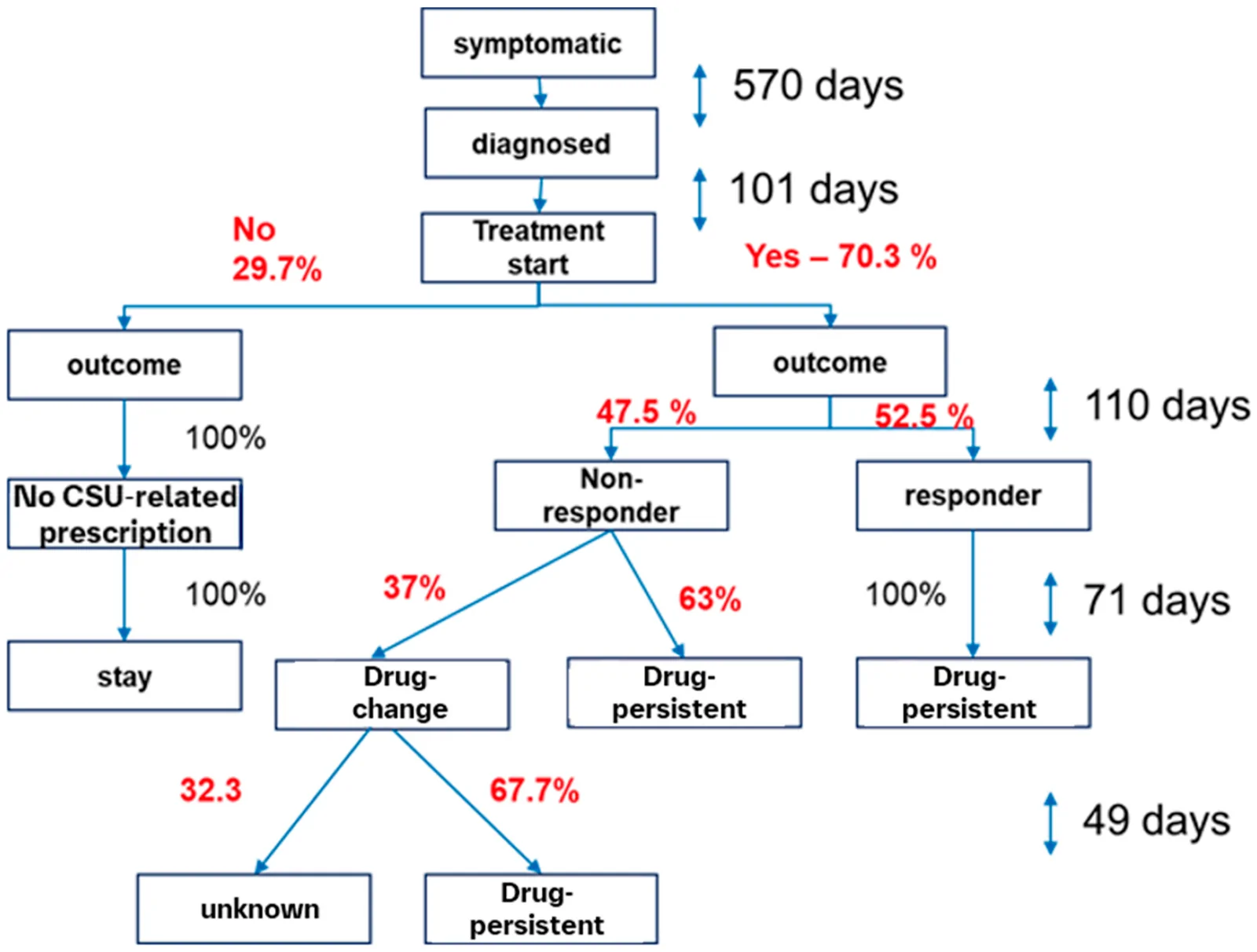
Derivation of patient journey model parameters from EHR data. The figure summarizes the sequential extraction of patient-level information from structured diagnoses, NLP-derived symptoms, and medication records. Observed proportions and time intervals were subsequently used to estimate transition probabilities and transition times for the Markov patient journey model. ‘no recorded CSU prescription’ shall be understood as a descriptive observation, not an end state.

**Figure 4:**
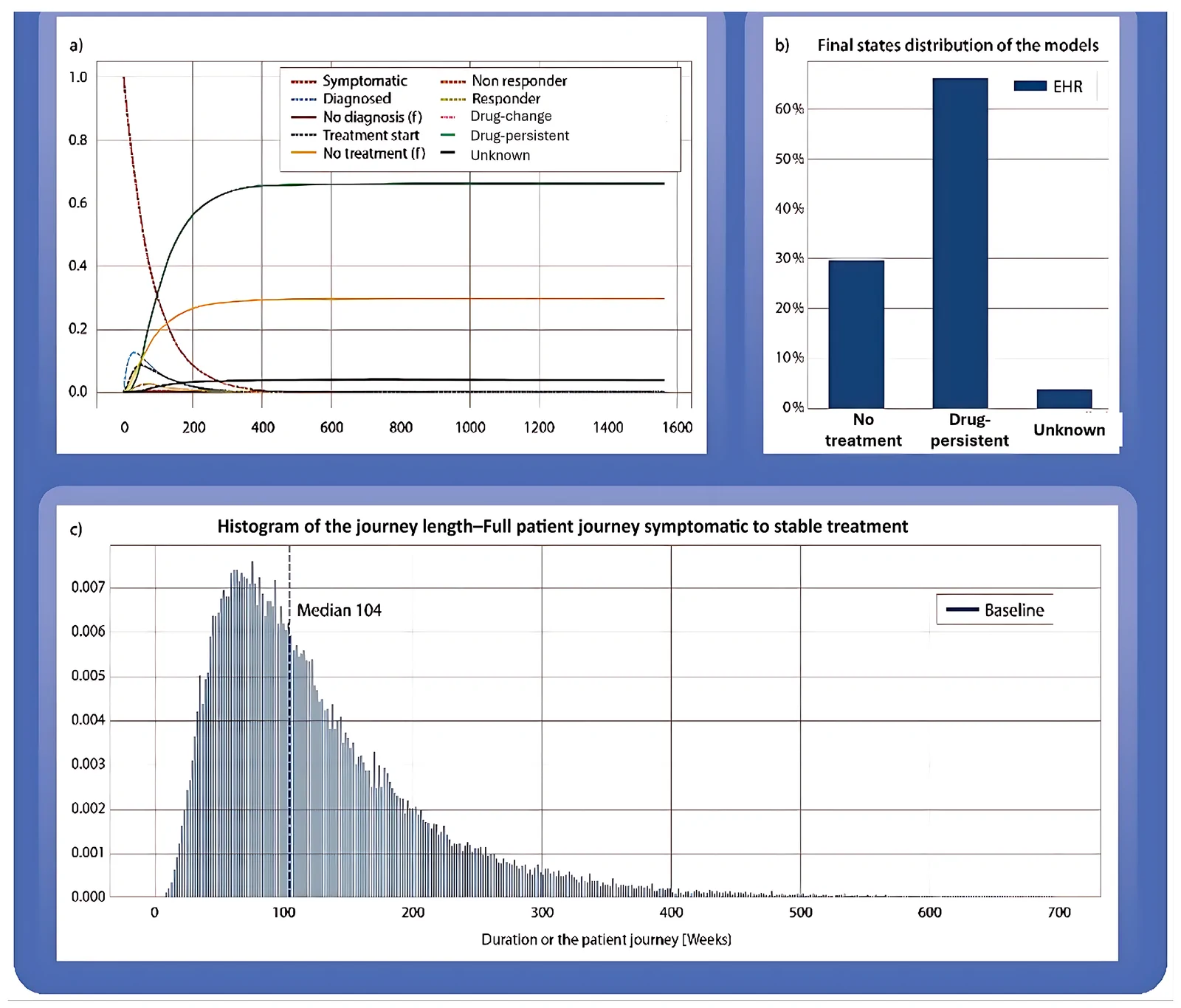
Data-driven reconstruction of the CSU patient journey using a Markov framework. Transition probabilities and time intervals estimated from OPTUM^®^ EHR data were incorporated into the Markov model to simulate patient movement across clinically relevant care steps derived from observed data. Panel (**a**) shows state occupancy over time, Panel (**b**) the distribution of patients across states at the end of follow-up, and Panel (**c**) the distribution of journey durations derived from observed transition times.

**Table 2:** Calculation of time to event using standard statistical analysis as calculation of mean, median and standard deviation of obtained data. Time is depicted as days and a value of time to event = 0 refers to 0 days between first documented symptom occurrence and diagnosis (in other words, symptom status and diagnosis were made on the same day) according to the definitions described above.

Parameter	n (Evaluable)	Same-Day Interval	No Documented Interval	Mean ± SD (Days)	Median (Days)
**Symptom to diagnosis**					
Structured ICD-9/10 symptom codes only	29,457	16,200 (55.0%)	45,207	157 ± 287	0
Structured codes plus NLP	56,551	6800 (12.0%)	18,113	570 ± 395	624
**Diagnosis to treatment**					
First CSU-related treatment	74,664	27,100 (36.3%)	22,200	101 ± 208	0
Second CSU-related treatment	3630	476 (13.1%)	0	194 ± 244	99
ThirdCSU-related treatment	8500	716 (8.4%)	65,400	282 ± 308	157
Fourth treatment	2900	118 (4.1%)	71,700	331 ± 326	209

Abbreviations: CSU, chronic spontaneous urticaria; ICD, International Classification of Diseases; NLP, natural language processing; SD, standard deviation. Values are days. n = patients with an evaluable interval; unless stated otherwise the denominator is the 74,664 diagnosed patients. Same-day interval = both events documented on the same date. These are documented events, not missing or placeholder values. No documented interval = no date recorded for one of the two events. These records were excluded from the respective estimate (missing, not zero). Mean ± SD refers to patients with an evaluable interval.

## Data Availability

The data used in this study are available from Optum^®^ (Optum^®^, Eden Prairie, MN, USA) but restrictions apply to their availability, as the data were obtained under license for the current study and are therefore not publicly available. Interested researchers may apply for access directly through Optum and must comply with applicable licensing and data use requirements.

## Supplementary Materials

The following supporting information can be downloaded at: https://www.mdpi.com/article/10.3390/healthcare14213768/s1, Figure S1: Schema showing the structure of the patient journey model as a discrete-state Markov framework.; Figure S2: Workflow to apply Natural language processing (NLP) for medical notes; Table S1: Applied variables within to extract unstructured data from EHR upon NLP technique; Table S2b: Search terms applied to identify CSU-related symptoms in clinical notes using the NLP functionality of the OPTUM® EHR database. Below the scope of the search is listed; Table S2b: Search terms applied to identify CSU-related symptoms in clinical notes using the NLP functionality of the OPTUM® EHR database. Below the individual search terms are listed; Table S3: Descriptive comparison of state proportions from EHR-derived patient journey parameters with estimates from the published CSU patient journey model by Maurer et al. (2024) [7]. intended as formal external validation; Table S4: Descriptive comparison of temporal sequence of transitions extracted from EHR-derived patient journey parameters with estimates from the published CSU patient journey model by Maurer et al. (2024) [7].
