# Supplementary material for: From Manual Search to Artificial Intelligence: A Methodological Framework for Developing Patient Journey Models from Electronic Health Records Using Natural Language Processing
Source: Healthcare (Basel). 2026 Nov 4;14(21):3768. doi: 10.3390/healthcare14213768 (PMC13762474; doi:10.3390/healthcare14213768)
Supplement: Supplementary file 1 [file healthcare-14-03768-s001.zip › healthcare-4644411-supplementary.pdf]

Supplementary material

From manual search to artificial intelligence: A methodological framework for developing patient journey models from electronic health records using natural language processing

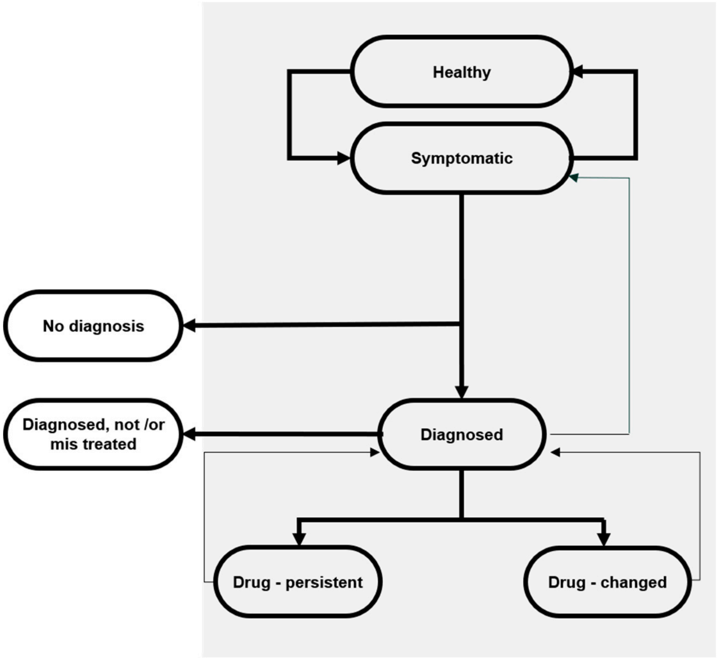

Fig.S1 Schema showing the structure of the patient journey model as a discrete-state Markov framework. The model contains five health states representing key stages of the disease pathway: Healthy, Symptomatic, Diagnosed, Drug-persistent and Drug-changed (grey area). Patients without a documented diagnosis or without recorded therapy are shown outside the modeled states; this reflects the absence of documentation in the EHR and does not imply absence of care. Explanation: *Drug-persistent*: a patient with at least one documented CSU-related prescription and no subsequent change in the prescribed drug (ATC/NDC code) during the observation period. *Drug-changed*: a patient with at least one change in the ATC/NDC code relative to the immediately preceding CSU-related prescription during the observation period.

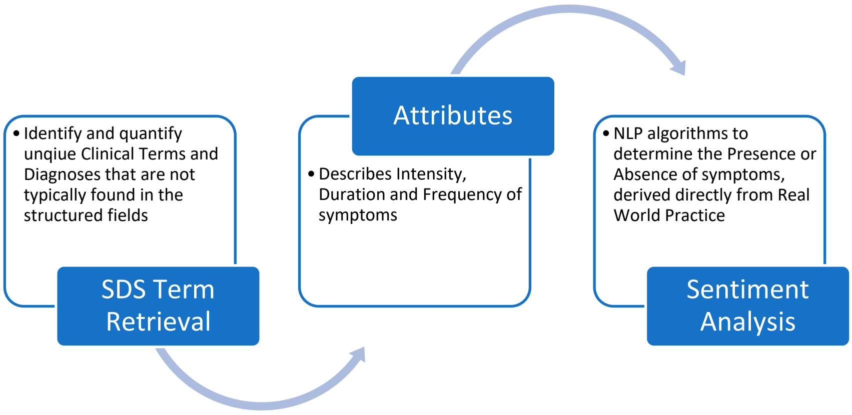

Fig.S2 Workflow to apply Natural language processing (NLP) for medical notes. SDS structured data storage

**Variable**	**Description**	**Example**
Term	Patient self-reported SDS	Exclude: *asthma, joint, tissue* etc<br>Include: *rash, sleep, itch* etc
Attribute	Qualitative descriptor of SDS indicating intensity	Exclude *allergen*
Sentiment	Indicates the presence or absence of a symptom or provide context for a reported term. Often conveys negation.	Exclude *negative, deny, rule out* etc
Location	Reported SDS term location found in clinical notes	Exclude *lung, shoulder,sinus* etc
Concept	Standardized and structured medical information extracted from free-text clinical notes	Exclude *pain*

Table S1 Applied variables within to extract unstructured data from EHR upon NLP technique.

Table S2a.

Search terms applied to identify CSU-related symptoms in clinical notes using the NLP functionality of the OPTUM® EHR database. Below the scope of the search is listed.

**Search category**	**Terms applied**	**CSU specificity**
Symptom terms	itch, rash, pruritus, wheals, pain, sleep problems	Only "wheals" is CSU-specific; the remaining terms are unspecific
Anatomical locations	skin, body, back, hands, face, feet, legs, arm, chest, neck	Generic; used to restrict findings towards CSU-typical sites, not as confirmation

Search category	Terms applied	CSU specificity
Search population	Patients with an ICD-10-diagnosed CSU in the cohort (n = 74,664)	Restricts the search to patients with CSU coding
Search period	Pre-diagnosis period only (before the index date)	Every additional mention can only precede the diagnosis

Table S2b.

Search terms applied to identify CSU-related symptoms in clinical notes using the NLP functionality of the OPTUM® EHR database. Below the individual search terms are listed.

Symptom terms	Anatomical location terms
itch	skin
rash	body
pruritus	back
wheals	hands
pain	face
sleep problems	feet
–	legs
–	arm
–	chest
–	neck

Notes. The search was applied only within the cohort of patients with an ICD-10-diagnosed CSU (n = 74,664) and only to the period before the index date. Anatomical location terms were used to restrict the findings towards CSU-typical sites; they do not confirm a CSU-specific aetiology. Negated findings were excluded. Assertion, historical and experiencer status were not available in the NLP output provided to the investigators, so mentions of other conditions or of earlier, unrelated episodes within the same patient record cannot be excluded. Only the term “wheals” is specific to CSU. The terms “pain” and “sleep problems” are not dermatological symptoms, and “itch”, “rash” and “pruritus” occur in many conditions. Additional symptom mentions identified by these terms therefore precede the diagnosis and lengthen the estimated symptom-to-diagnosis interval; they cannot shorten it.

Table S3: Descriptive comparison of state proportions from EHR-derived patient journey parameters with estimates from the published CSU patient journey model by Maurer et al. (2024) [7]. intended as formal external validation.

**Parameter**	**Maurer et al. (2024) [7]**	**Present work (EHR)**	**Operational definition in the present work**
Overall treatment rate	65.3%	70.3%	At least one documented CSU-related prescription after the index date
Patients with a first treatment change	25%	47.5%	Change in the ATC/NDC code relative to the preceding CSU-related prescription; assessed at drug level, so a change within the same therapeutic class counts
Drug-persistent after the 1st prescription	38.6%	52.5%	At least one documented CSU-related prescription with no subsequent change in the prescribed drug
Drug-changed after the 2nd prescription	37.8%	37%	Change in the ATC/NDC code following the second documented CSU-related prescription
Drug-persistent after the 2nd prescription	63.2%	63%	No change in the prescribed drug following the second documented prescription
Drug-persistent after the 3rd prescription	67%	67.7%	No change in the prescribed drug following the third documented prescription
Drug-changed after the 3rd prescription	33%	32.3%	Change in the ATC/NDC code following the third documented prescription

Table S4: Descriptive comparison of temporal sequence of transitions extracted from EHR-derived patient journey parameters with estimates from the published CSU patient journey model by Maurer et al. (2024) [7].

Transition	Maurer et al. (2024) [7]	Present work (EHR)	Definitions and notes
Symptomatic to diagnosed	2 years	570 days (codes + NLP) 157 days (codes only)	Two definitions. 570 days: earliest positive symptom mention from ICD-9/10 codes and NLP, likely an upper estimate because mentions are not CSU-confirmed and historical mentions cannot be excluded. 157 days: symptoms identified from ICD-9/10 codes only, a lower estimate. Median 624 and 0 days, respectively.
Diagnosis to first prescription	3 months	101 days (median 0)	First documented CSU-related prescription after the index date. 27,100 of 74,664 patients had a same-day interval and 22,200 had no documented interval.
Second treatment	20 weeks	194 days (median 99)	Second documented CSU-related prescription; omalizumab subgroup n = 3,630.
Third treatment	32 weeks	282 days (median 157)	Third documented CSU-related prescription; 716 patients with a same-day interval, 65,400 without a documented interval.

Notes. The comparison is descriptive and is not intended as formal external validation. The two data sources differ in several respects: the comparison model was built from expert-based and literature-derived inputs, whereas the present values are derived from documented prescribing events in a US EHR database. Treatment states in the present work are prescribing-pattern states and not clinical disease states. A change in the prescribed drug was defined as an ATC/NDC code different from the immediately preceding CSU-related prescription and was assessed at the level of the individual drug rather than the drug class, because cycling within a therapeutic class is common in CSU
